# Survey-based data describing readiness to adopt an electronic pregnancy registration-monitoring system amongst health workers

**DOI:** 10.1016/j.dib.2020.106192

**Published:** 2020-08-18

**Authors:** Sandra Hakiem Afrizal, Achmad Nizar Hidayanto, Putu Wuri Handayani, Kemal Nazaruddin Siregar, Besral, Meiwita Budiharsana, Tris Eryando, Nashrul Hakiem

**Affiliations:** a Faculty of Health Science, Universitas Esa Unggul, Jakarta, Indonesia; b Faculty of Computer Science, Universitas Indonesia, Depok, Indonesia; c Faculty of Public Health, Universitas Indonesia, Depok, Indonesia; d Faculty of Science and Technology, Universitas Islam Negeri Syarif Hidayatullah, Jakarta, Indonesia

**Keywords:** Electronic health, Primary health care, Readiness, Midwife, Community health worker, Monitoring System

## Abstract

Electronic Health (eHealth) systems show a growing trend in developing countries to enhance their respective healthcare services. However, there is a lack of empirical study regarding readiness during preparation for eHealth implementation in Primary Health Care (PHC) units, specifically for antenatal care health workers who serve not only for personal care but also community services. The survey-based data applied in this research describes the assessment of the pre-implementation of the Electronic Pregnancy Registration and Monitoring System amongst health workers who involved in ANC services in primary health care (PHC) units of South Tangerang district, an urban area of Banten Province, Indonesia. Primary data was collected from 210 ANC health workers who work in 6 PHCs of the district. The data consists of socio-demographic factors of respondents such as age, education, years of experience etc., and captures individual responses to measure their readiness for eHealth adoption. The availability of this data will provide valuable information for researchers, healthcare organisations and government as the policy makers to prepare strategies with regard to readiness for eHealth adoption amongst health workers in PHCs

**Specifications Table**

**Table:** 

**Subject**	Health profession, health information management
**Specific subject area**	Preparedness for electronic pregnancy monitoring system implementation amongst health workers
**Type of data**	Table
**How data were acquired**	Individual-based questionnaire based on convenience sampling from respondents who are involved in the ANC registration and monitoring process
**Data format**	Raw and table
**Parameters for data collection**	Socio-demographic aspect of individual readiness amongst health workers with regard to electronic pregnancy monitoring system implementation
**Description of data collection**	A questionnaire was distributed to evaluate the Independent variables for readiness which were developed based on socio-demographic aspects. The questionnaire is provided as a supplementary file
**Data source location**	Public Primary Health Care, South Tangerang District, Banten Province, Indonesia
**Data accessibility**	Repository name: Mendeley Data Data identification number: 10.17632/p67yb9z8ph.2 Afrizal, SH (2020), “Dataset of determinants to health workers readiness towards e-pregnancy monitoring system”, Mendeley Data, v2, http://dx.doi.org/10.17632/p67yb9z8ph.2 [1]

**Value of the Data**

• The datasets provide an insight into the association of socio-demographic factors amongst health workers with individual preparedness before implementing eHealth such as the electronic monitoring system for pregnancy.
• This data is useful for all levels of health care organisation as well as the health workers namely midwives and community health cares for further intervention to enhance the adoption of an electronic monitoring system for pregnancy.
• This data can be used as valuable information for creating the local and the central government awareness of the of the benefits of e-Health on monitoring system [2,3], improving technology knowledge [4], and increasing willingness [5] to enhance the antenatal care quality through e-pregnancy monitoring system implementation amongst the health workers in emerging technologies.
• The dataset can be implied for the other regions through the understanding of readiness to adopt an electronic monitoring system.

## 1 Data description

**Fig. 1:**
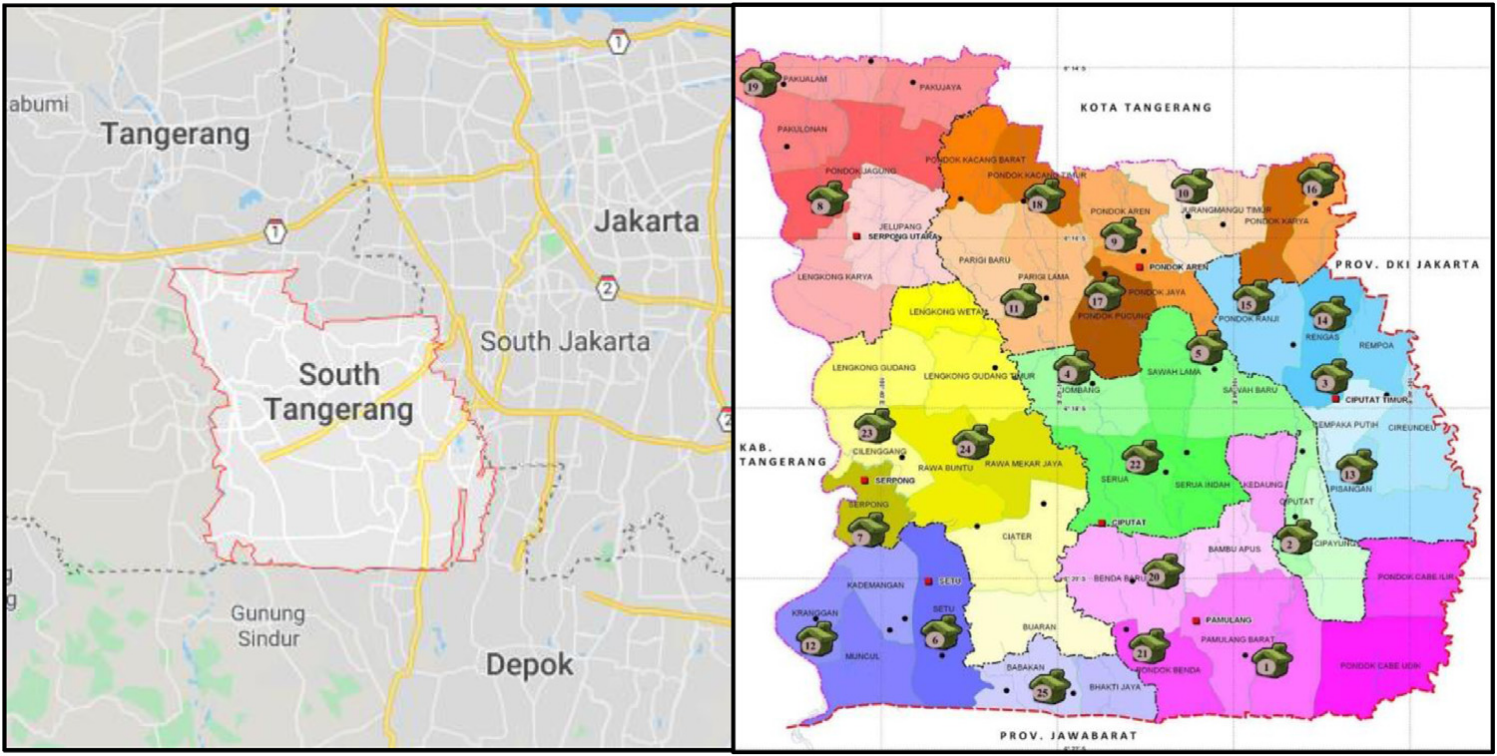
Left picture: South Tangerang district, the urban area of Banten Province-Indonesia. Right picture: distribution of primary health cares in the district.

Sources: Google Maps and District Health Office Profile

This survey data delivers information concerning the current implementation performed during the antenatal care service in primary health care and the factors related to readiness to adopt an electronic monitoring system. Specifically, the electronic Pregnancy Registration and Monitoring System, as applied amongst antenatal care providers in Primary Heath Care of South Tangerang District of Banten Province, Indonesia (see Fig. 1).

The dataset consists of information about the perceptions of providers regarding IT adoption readiness. It contains demographic data of the respondents such as age, education level, job description, and working experience. It also provides the social data which relates their behaviour with regard to preparedness for eHealth implementation such as hand phone ownership, previous experience in health information systems, knowledge of use of a computer, accessing the Internet, using social media applications, providing a monthly budget for the Internet, using a hand phone for communication, and finally willingness to use a new innovation [1].

Table 1 and Table 2 describe the distribution of respondents and social-demographic variables in detail which can be used by future researchers working in the same topic. For a normal pregnancy, antenatal care is performed in primary health care (PHC) at least four times whereby the first visit is in the first trimester, the second visit is in the second trimester, and the last two visits are completed in the third trimester [6,7]. The antenatal care can also be undertaken through home visits or in a satellite PHC visit especially when pregnant women are not able to visit the PHC [8]. The integration of care between in-house and the community is an anticipatory step to prevent the occurrence of maternal deaths caused by inadequate examination.

**Table 1:** Distribution of Health workers as respondents in South Tangerang.

Sub District	Frequency	Total (%)
Ciputat	24 Midwives, 40 CHWs	64 (30.5)
Ciputat Timur	22 Midwives, 37 CHWs	59 (28.1)
Pamulang	12 Midwives, 41 CHWs	53 (25.2)
Pondok Aren	12 Midwives, 22 CHWs	34 (16.2)
Total	210	(100)

CHWs: Community Health Workers.

**Table 2:** Socio-demographic profile of the respondents (*n* = 210) and association to readiness.

Variables	Category	Readiness		Total (%)
		Ready n (%)	Not Ready n (%)	
Age	≤ 25 years	13 (100)	–	13 (6.2)
	26 to 35	40 (93)	3 (7)	43 (20.5)
	36 to 45	61 (85.9)	10 (14.1)	71 (33.8)
	≥ 46	74 (89.2)	9 (10.8)	83 (39.5)
Education Level	Primary and Secondary	103 (85.1)	18 (14.9)	121 (57.6)
	Diploma	67 (94.4)	4 (5.6)	71 (33.8)
	Degree & Post Graduate	18 (100)	–	18 (8.6)
Working experience	≤ 5 years	41 (91.1)	4 (8.9)	45 (21.4)
	6 to 10	68 (84)	13 (16)	81 (38.6)
	11 to 20	56 (96.6)	2 (3.4)	58 (27.6)
	≥ 21	23 (88.5)	3 (11.5)	26 (12.4)
Smartphone ownership	Yes	185 (89.4)	22 (10.6)	207 (98.6)
	No	3 (100)	–	3 (1.4)
Experience in HIS Implementation	Yes	82 (92.1)	7 (7.9)	89 (42.4)
	No	106 (87.6)	15 (12.4)	121 (57.6)
Computer Application Knowledge	Good	115 (93.5)	8 (6.5)	123 (58.6)
	Limited	73 (83.9)	14 (16.1)	87 (41.4)
Access to Internet	Good	47 (88.7)	6 (11.3)	53 (25.2)
	Limited	141 (89.8)	16 (10.2)	157 (74.8)
Social Media Apps in Handphone	Yes	175 (92.1)	15 (7.9)	190 (90.5)
	No	13 (65)	7 (35)	20 (9.5)
Monthly Internet Budget	Yes	154 (89.5)	18 (10.5)	172 (81.9)
	No	34 (89.5)	4 (10.5)	38 (18.1)
Use of HP for care-coordination	Yes	130 (91.5)	12 (8.5)	142 (67.6)
	No	58 (85.3)	10 (14.7)	68 (32.4)
Willingness to use App	Yes	129 (94.9)	7 (5.1)	136 (64.8)
	No	59 (79.7)	15 (20.3)	74 (35.2)
Job Title	Midwife	66 (94.3)	4 (5.7)	70 (33.3)
	CHW	122 (87.1)	18 (12.9)	140 (66.7)

Based on the Indonesian Ministry of Health regulations, integrated antenatal care is defined as serial health-care for a woman that starts from conception until delivery of the baby [7]. Prior to that, an integrated antenatal care process for pregnant women in Primary Health Care is a form of comprehensive care performed by health workers which is led by the midwives and assisted by the community health workers (CHWs) in several steps such as: 1) providing effective communication regarding a woman's condition, nutritional status, risk assessment; 2) early risk detection and assessment; 3) safe birth preparedness; 4) case management and referral system; and 5) family support.

As the Ministry of Health released a new regulation in early 2019 regarding the minimum standard for ANCs that should be obtained by all pregnant mothers in each PHC coverage area, it becomes necessary for the health workers to improve their work. Previous study has concluded that most of the midwives in Indonesia are well-trained in manual registration-monitoring system, however, most of them are unfamiliar with the electronic application [9]. Similar research conducting by Afrizal, et.al, 2019 in South Tangerang, it is found that the manual registration and monitoring system during the antenatal care visit have barriers of causing in the inadequate documentation. The ineffective working process such as various kinds of paper-based records has increased the workload and resulted in incomplete documentation although they have enough knowledge in the registration-monitoring process [10]. Researchers [2,3] have studied that effective and efficient care-coordination can made possible by utilising technology such as an electronic system for monitoring and registration purposes.

## 2 Experimental design, materials, and methods

The population of midwife and community health workers in Primary Health Care were estimated to be 5500 in the South Tangerang District [11]. The sample size was calculated using a sample size calculator [12] with a confidence level of 95%. The proportion based on previous studies was 12% [13] with a margin of error of 5%. The sample size should be 169 participants accordingly. This figure included midwives and community health workers as the first line of ANC service and to provide the ANC documentation in the Primary Health Care units. Hence, it was possible to distribute 250 questionnaires which was a number higher than the proposed number of respondents.

For conducting the field survey, the convenience sampling method was adopted. The items in the survey were written using the Indonesian language to obtain the perspectives of the respondent. Altogether, around 250 health workers (100 midwives and 150 CHWs) were approached. Amongst these, questionnaires from 40 respondents were discarded as they were incompletely completed. Finally, the data was collected for 210 respondents (response rate 84%).

The dependant variable was readiness (this research was evaluated in different sections of the study). To assess the level of readiness the authors modified and delivered 20 questions developed by Aydin, et al., 2005. Based on the prior research, the options of the respondent answers can easily be coded as 1, 2, 3, 4, and 5, as in a five-point Likert-scale. The mean score 3.4 can be identified as the middle point level of readiness where the categorisation of ‘ready’ applies if the mean >3.4 and ‘not ready’ if the mean ≤ 3.4 [14]. With regard to the classification, based on an unpublished study by the authors, 22 (10.4%) of the respondents to this research were categorised as “not ready” while 188 (89.5%) respondents were categorised as “ready”. The next step is selection of the independent variables using bivariate test to show the association. Table 2 provides the data collected from the health workers of six public PHCs.

The dataset provides a comprehensive point of view regarding to the socio-demographic characteristics of the health workers. Majority of the respondents are in the age group of ≥46 (36.5%) respectively. However, we can observe that, more than 93% of the respondents in the age group of <35 are ready to adopt an electronic system. Thus, it can be inferred that the people at this age group can be easily trained to implement the electronic pregnancy monitoring application with minimal efforts.

Furthermore, the data shows that approximately 57% of the respondents have education beyond secondary school. However, respondents who have diploma and higher education are likely ready and may not find trouble in understanding the electronic pregnancy monitoring application since the percentage of readiness is more than 94% amongst the respondents. For the adoption of e-Health services, it is important to have access to internet and monthly internet budget. Data shows that around 81.9% of the respondents have monthly internet budget of which 89.5% to have readiness to implement an e-pregnancy monitoring system. Only 25.2% of the respondents claim to have access to internet in the working place which may cause barriers in adopting a new technology. The limitation to internet access gives opportunity to the health organisations to improve the infrastructure in terms of internet sources in the Public Primary Health Care to improve the e-health services.

The data shows that 98.6% of the respondents have a smartphone and 90.5% have a social media application in their phone. The increased usage of smartphone amongst the health workers gives an opportunity for local and national government as the policymakers to explore the feature of mobile monitoring application and provide training to use smartphone amongst health workers. It was interesting to see that 64.8% of the respondents were interested in IT adoption for pregnancy monitoring system with 94% showing their willingness to use an electronic pregnancy monitoring system during the ANC process. Finally, the above-mentioned dataset has the potential to expose several interesting findings to accelerate the adoption of e-pregnancy monitoring system amongst health workers through future research in this area. Although this dataset represents the urban area health workers, the trend is similar across the urban area in Indonesia to face the disruption of information technology.

### 2.1 Ethical considerations

The study was only performed after receiving formal clearance number 783/UN2.F10/PPM.00.02/2018 from the ethical board of Universitas Indonesia. The background and aim of this research were explained to the respondents.

### 2.2 Theoretical, practical and policy implications of the data article

The current data article provides theoretical implications regarding factors associated with the preparedness for the adoption of an ANC monitoring and registration system from the perspective of the health workers. The socio-demographic profiles and technology preparedness of the midwives and community health services as the first responder in the ANC process in primary health care should be taken as a recommendation and a plan for improving the IT adoption uptake. These findings are also important for practical implication with regard to the preparedness for implementation of the electronic Pregnancy Registration and Monitoring System. The other part which lacking attention is personal data protection. Future works should be performed as consideration since this could potentially improve the adoption of electronic record system, starting from the root of the health organisation such as Primary Health Care as well as the Ministry of Health in terms of policy implications. It is necessary to pay more attention to the abovementioned variables which may be used to generate new policies regarding eHealth adoption for a maternal monitoring system.

## Declaration of Competing Interest

The authors declare that they have no known competing financial interests or personal relationships which have, or could be perceived to have, influenced the work reported in this article.

## Appendix Supplementary materials

Image, application 1

Image, application 2
