# Supplementary material for: Survey-based data describing readiness to adopt an electronic pregnancy registration-monitoring system amongst health workers
Source: Data Brief. 2020 Aug 18;32:106192. doi: 10.1016/j.dib.2020.106192 (PMC7452415; doi:10.1016/j.dib.2020.106192)
Supplement: Supplementary file 2 [file mmc2.docx]

**SUPPLEMENTARY FILE**

**“QUESTIONNAIRE OF SOCIO-TECHNICAL READINESS ASSESSMENT TOWARDS AN ELECTRONIC PREGNANCY REGISTRATION-MONITORING SYSTEM IMPLEMENTATION IN PUBLIC PRIMARY HEALTH CARE”**

**2019**

**CODE : B……..**

1. **RESPONDENT IDENTITY**

Name : ………………………………………………………

Address : ………………………………………………………

1. **INSTRUCTIONS**

An Electronic Pregnancy Monitoring and Registration System is an application which will be used by midwives and Community Health Workers (CHW) to monitor all of pregnant women in the Public Primary Health Care coverage area. This application can be accessed using a smart phone for user convenience. This survey aims to assess the readiness of midwives and CHW before adopting a new system.

1. Please give a cross sign ⮽ for your best individual answer
2. In section I, please give your response to the items
3. In section II, please rate the aspect of readiness to implement an electronic pregnancy monitoring-registration system on a scale:

1 = “Strongly Disagree”

2 = “Disagree”

3 = “Neutral or neither agree nor disagree”

4 = “Agree”

5 = “Strongly Agree”

| - 1. **DETERMINANT FACTORS** | | | | | |
| --- | --- | --- | --- | --- | --- |
| 1. Age | ( ) Years | | | | |
| 1. Working Experience | 🞎 ≤ 5 Years 🞎 11-20 Years  🞎 6-10 Years 🞎 ≥ 21 Years | | | | |
| 1. Education | 🞎 Primary/Secondary/High School  🞎 Diploma  🞎 Bachelor Degree  🞎 Post Graduate | | | | |
| 1. Job Position | 🞎 Midwife  🞎 Community Health Worker | | | | |
| 1. Computer Application Knowledge   Write down the answer by choosing one of the right answer from the next column:   1. Electronic applications used to write letters or messages 2. Benefits of health applications for Primary Health Care 3. Application used to create document or note 4. Health Information System for Public Primary Health Care in Indonesia 5. Health records for patients using electronic devices | 1. To improve patient safety 2. SIKDA 3. Electronic medical record 4. To prevent disaster 5. Power Point 6. Ms. Word 7. E-mail 8. E-Puskesmas | | | | |
| 1. Do you have a Smartphone? (a mobile phone that includes advanced functionality beyond making phone calls and sending text messages such as send e-mail, chat) 2. Do you have a social media such as Facebook, Instagram, or Twitter | 🞎 Yes 🞎 No  🞎 Yes 🞎 No | | | | |
| 1. Do you have Experience in Health Information System (HIS) Implementation | 🞎 Yes 🞎 No | | | | |
| 1. Do you have access to internet in your working place? 2. Do you have monthly internet budget? 3. Do you prefer to use your handphone to communicate or coordinating the patient’s care by sending SMS or WhatsApp? 4. Are you willing to use an electronic pregnancy monitoring and registration system? | 🞎 Yes 🞎 No  🞎 Yes 🞎 No  🞎 Yes  🞎 No, I prefer to use a manual book  🞎 Yes 🞎 No | | | | |
| - 1. **READINESS ASSESSMENT** | 1. **Strongly Disagree** | 1. **Disagree** | 1. **Neutral** | 1. **Agree** | 1. **Strongly Agree** |
| 1. Most of employees are experienced in using an e-Health system | 🞎 🞎 🞎 🞎 🞎 | | | | |
| 1. The management of PHC organization has declared to use the electronic health information system in a daily task | 🞎 🞎 🞎 🞎 🞎 | | | | |
| 1. IT officer assistance for e-Health system is available in the organisation | 🞎 🞎 🞎 🞎 🞎 | | | | |
| 1. Previous experience in implementing an electronic health information system in the organization has improved the efficiency in monitoring system | 🞎 🞎 🞎 🞎 🞎 | | | | |
| 1. Most of the health workers are willing to implement the Electronic Pregnancy Registration-Monitoring System | 🞎 🞎 🞎 🞎 🞎 | | | | |
| 1. Based on previous experience, the electronic health information system is able to improve the process of coordination of care between professions | 🞎 🞎 🞎 🞎 🞎 | | | | |
| 1. Monitoring from the organization management is carried out during the adoption of an innovation (such as the Electronic Pregnancy Registration-Monitoring System) | 🞎 🞎 🞎 🞎 🞎 | | | | |
| 1. Legal issue such as punishment is able to reduce the barrier of the adoption of an innovation in the organisation (such as the Electronic Pregnancy Registration-Monitoring System) | 🞎 🞎 🞎 🞎 🞎 | | | | |
| 1. Reward is able to increase the adoption of an innovation in the organization | 🞎 🞎 🞎 🞎 🞎 | | | | |
| 1. Internet access is available at the PHC to support the implementation of an Electronic Pregnancy Registration-Monitoring System | 🞎 🞎 🞎 🞎 🞎 | | | | |
| 1. PHC has achieved the organizational change target (such as total quality indicators) after an innovation implementation such as an electronic health information system | 🞎 🞎 🞎 🞎 🞎 | | | | |
| 1. Officers who can facilitate the technology implementation will help you to implement the Electronic Pregnancy Registration-Monitoring System | 🞎 🞎 🞎 🞎 🞎 | | | | |
| 1. You have access to use computer individually at work | 🞎 🞎 🞎 🞎 🞎 | | | | |
| 1. Based on previous experience, the electronic health information system is able to save the patience personal data securely | 🞎 🞎 🞎 🞎 🞎 | | | | |
| 1. Your computer has ability to create, save, delete and protect the file | 🞎 🞎 🞎 🞎 🞎 | | | | |
| 1. You can follow the direction on a computer screen to accomplish a task in an application | 🞎 🞎 🞎 🞎 🞎 | | | | |
| 1. Electronic Pregnancy Registration-Monitoring System as an innovation will integrate the health care service for community and personal health care | 🞎 🞎 🞎 🞎 🞎 | | | | |
| 1. Electronic Pregnancy Registration-Monitoring System as an innovation will improve the record completeness and easy to access | 🞎 🞎 🞎 🞎 🞎 | | | | |
| 1. Electronic Pregnancy Registration-Monitoring System as an innovation will improve the timely antenatal care visit for pregnant mother | 🞎 🞎 🞎 🞎 🞎 | | | | |
| 1. Electronic Pregnancy Registration-Monitoring System as an innovation meets the organisation’s needs to change the previous manual monitoring system | 🞎 🞎 🞎 🞎 🞎 | | | | |

THANK YOU FOR YOUR PARTICIPATION
